# Fluid overload in children undergoing mechanical ventilation

**DOI:** 10.5935/0103-507X.20170045

**Published:** 2017

**Authors:** Clarice Laroque Sinott Lopes, Jefferson Pedro Piva

**Affiliations:** 1 Postgraduate Program in Child and Adolescent Health, Universidade Federal do Rio Grande do Sul - Porto Alegre (RS), Brazil.; 2 Pediatric Intensive Care Unit, Hospital de Clínicas de Porto Alegre - Porto Alegre (RS), Brazil.

**Keywords:** Water-electrolyte imbalance, Respiration, artificial, Renal insufficiency, Fluid therapy, Hemodynamics

## Abstract

Patients admitted to an intensive care unit are prone to cumulated fluid overload and receive intravenous volumes through the aggressive resuscitation recommended for septic shock treatment, as well as other fluid sources related to medications and nutritional support. The liberal liquid supply strategy has been associated with higher morbidity and mortality. Although there are few prospective pediatric studies, new strategies are being proposed. This non-systematic review discusses the pathophysiology of fluid overload, its consequences, and the available therapeutic strategies. During systemic inflammatory response syndrome, the endothelial glycocalyx is damaged, favoring fluid extravasation and resulting in interstitial edema. Extravasation to the third space results in longer mechanical ventilation, a greater need for renal replacement therapy, and longer intensive care unit and hospital stays, among other changes. Proper hemodynamic monitoring, as well as cautious infusion of fluids, can minimize these damages. Once cumulative fluid overload is established, treatment with long-term use of loop diuretics may lead to resistance to these medications. Strategies that can reduce intensive care unit morbidity and mortality include the early use of vasopressors (norepinephrine) to improve cardiac output and renal perfusion, the use of a combination of diuretics and aminophylline to induce diuresis, and the use of sedation and early mobilization protocols.

## Abstract (pt)

Os pacientes admitidos em uma unidade de terapia intensiva estão sujeitos à sobrecarga fluídica acumulada e recebem volume endovenoso pela ressuscitação agressiva, preconizada nas recomendações de tratamento do choque séptico, além de outras fontes de líquidos relacionadas às medicações e ao suporte nutricional. A estratégia liberal de oferta hídrica tem sido associada a maiores morbidade e mortalidade. Apesar de haver poucos estudos prospectivos pediátricos, novas estratégias estão sendo propostas. Esta revisão não sistemática discute a fisiopatologia da sobrecarga fluídica, suas consequências e as estratégias terapêuticas disponíveis. Durante a síndrome da resposta inflamatória sistêmica, o glicocálice endotelial é danificado, favorecendo o extravasamento fluídico, traduzido em edema intersticial. O extravasamento para o terceiro espaço se traduz em maior tempo de ventilação mecânica, maior necessidade de terapia de substituição renal e mais tempo de internação na unidade de terapia intensiva e no hospital, entre outros. A monitorização hemodinâmica adequada, bem como a infusão cautelosa de fluídos, pode minimizar estes danos. Uma vez instalada a sobrecarga fluídica acumulada, o tratamento com o uso crônico de diuréticos de alça pode levar a uma resistência ao uso destas medicações. A utilização precoce de vasopressores (norepinefrina) para melhora do débito cardíaco e perfusão renal, a associação de diuréticos e uso da aminofilina para indução de diurese, e a utilização de protocolos de sedação e mobilização precoce são algumas estratégias que podem reduzir morbimortalidade na unidade de terapia intensiva.

## INTRODUCTION

The importance of fluid resuscitation for patients in shock and with systemic inflammatory response syndrome (SIRS) is indisputable, with an impact that reduces mortality and morbidity.^(1,2)^ However, new evidence shows that after initial management with intravenous fluids, fluid overload (FO), which frequently occurs in patients admitted to intensive care units (ICU), has deleterious effects and may lead to unfavorable outcomes, such as longer mechanical ventilation (MV), prolonged hospitalization, the need for renal replacement therapy (RRT), and higher mortality risk.^(3-5)^

## PATHOPHYSIOLOGY

**Figure 1:**
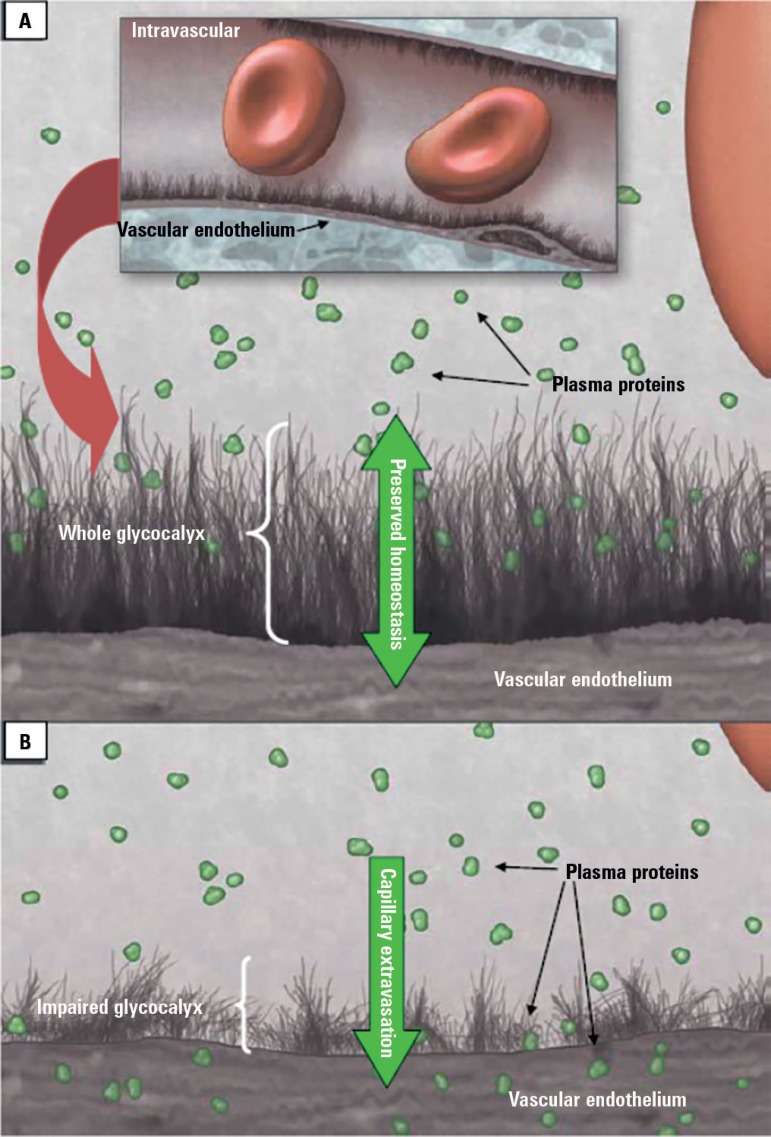
Schematic representation of the endothelial glycocalyx. Panel A shows a healthy glycocalyx maintaining transcapillary equilibrium; Panel B shows a damaged endothelial glycocalyx due to inflammatory process, such as sepsis, with extravasation occurring along with the development of edema and invasion of adjacent tissues by pro-inflammatory cytokines. Adapted from: Myburgh JA, Mythen MG. Resuscitation fluids. N Engl J Med 2013; 369 (13): 1243-51.^(7)^

The vascular endothelium allows the free passage of water, electrolytes, glucose, and nutrients. This transcapillary exchange depends on an optimal balance between hydrostatic pressure, which is determined by the intravascular volume, the endothelial tone, and the oncotic pressure attributed to proteins and colloids that remain in the intravascular space. The fluid that passes an intact vascular barrier through the intravascular and extravascular spaces is reabsorbed by the lymphatic system, making it impossible to develop edema.^(6)^ However, when inflammatory processes cause damage, the endothelial glycocalyx barrier breaks down, causing fluid extravasation and, as the process progresses, edema (Figure 1).^(7)^

In addition to the capillary structure, stable intravascular volume is also maintained by a sensitive and efficient feedback system by the baroreceptors located in the carotid sinus, atrium, and renal afferent arterioles. In response to any change in intravascular volume, the renin-angiotensin-aldosterone system is activated and natriuretic peptides are released, causing the retention of sodium and water at the renal level in an attempt to restore blood volume.^(8)^ The imbalance between Starling's forces and endothelial glycocalyx injury results in a transfer of intravascular fluid to the interstitium, resulting in edema, ascites, pleural effusion, and leakage into the third space. The relative decrease in circulating volume leads to hypotension, tissue hypoperfusion, and organ dysfunction.^(6,8,9)^

Several studies use the FO percentage (FO%) as a tool to estimate the amount of fluid retained relative to body weight and to verify its association with unfavorable outcomes. The FO percentage is calculated using the following formula:

**Table:** 

FO% = [(fluids administered - fluids eliminated)/weight at admission] x 100

Fluids are expressed in liters and the weight in kilograms.

Values of FO% ≥10% are strongly associated with higher morbidity, such as worse oxygenation levels, longer MV time, longer ICU stay, greater need for RRT, and even higher mortality.^(10-12)^

Acute kidney injury (AKI) is a known complication in patients admitted to an ICU. The medications used to manage shock can cause direct or indirect kidney injury, leading to worsening of kidney function and reduction of glomerular filtration; consequently, FO increases, which in turn may promote an increase in venous pressure, leading to increased renal subcapsular pressure, worsening renal perfusion, and a lower glomerular filtration rate.^(11,13)^ In developed countries, the most frequent risk factors for AKI are cardiac surgery, acute tubular necrosis, sepsis, and the use of nephrotoxic drugs.^(14)^

The epidemiology of renal failure in the ICU is unknown, but some studies estimate that the incidence may reach approximately 25% of critically ill children.^(15)^ Patients receiving RRT may present a mortality rate of 38 to 58%. One of the associated risk factors is the FO%; an FO% that reaches 10 to 20% has been shown to be an independent predictive factor for mortality.^(14)^

Post-operative cardiac studies demonstrated AKI in up to 45% of cases, with risk factors of age, use of cardiac bypass, cardiac surgery classification (Risk Adjustment For Congenital Heart Surgery - RACHS-1), need for vasopressors, and post-operative hypotension.^(16)^ Recently, a retrospective cohort involving 435 neonates receiving cardiac bypass surgery demonstrated that FO is an independent risk factor for unfavorable outcomes in the post-operative period and may be a non-invasive marker of kidney function.^(4,11,16)^ FO directly influences mortality, and it is estimated that for every 1% increase in FO%, there is a 36% increase in the mortality odds ratio.^(4)^

In children with acute respiratory distress syndrome (ARDS), early FO was associated with mortality and time of MV.^(17,18)^ A prospective observational study found that a positive water balance (water retention) during an ICU stay was associated with a higher mortality at 28 days.^(10)^ Another study evaluated respiratory morbidity and mortality in pediatric patients admitted to an ICU. In this study, FO was associated with worsening of the oxygenation level and with the highest number of days on invasive MV, but the study did not find an increase in mortality at 48 hours associated with FO.^(18)^ A retrospective study, which reviewed the oxygenation index, the FO%, and the daily pediatric logistic organ dysfunction prognostic index of 80 patients, also showed that peak FO% and FO severity are associated with a longer invasive MV time and a longer ICU stay.^(19)^

FO, manifested as interstitial edema and extravasation to the third space, is associated with impairment of the myocardium, central nervous system, hepatic function, and digestive system with nutrient malabsorption syndrome, thus triggering malnutrition, poor healing of wounds, and a higher risk of intra-abdominal hypertension and abdominal compartment syndrome.^(5,20)^

Patients on post-operative, trauma victims and those with ARDS who were subjected to different fluid resuscitation strategies presented higher morbidity and mortality that was associated with liberal fluid strategies.^(21-24)^ Patients with high FO% had more frequent dysfunction of multiple organs and death.^(25)^

## COMPOSITION OF RESUSCITATION FLUIDS

There is still no ideal fluid for the resuscitation of patients in shock. In addition to good cost-effectiveness, the fluid should have a chemical composition similar to that of plasma and reverse the signs of shock without extravasation into the extravascular space.^(26)^ Currently, two groups of fluids are available: crystalloids and colloids.

Crystalloids are recommended as the first line of fluids in reversing hemodynamic instability in patients in shock.^(1,6,27)^ These solutions are composed of ions with variable tonicity that can be distributed freely through the endothelial barrier. Saline solution is isotonic relative to plasma but has higher chloride concentrations, thus predisposing patients to hyperchloremic metabolic acidosis.^(28)^ The evidence for the influence of hyperchloremic metabolic acidosis on patient outcome remains unclear, but some studies have associated an increased risk of developing renal failure.^(26)^ The balanced solutions, Ringer's and Hartmann's solutions, are more hypotonic than extracellular fluid and are also associated with hyperchloremia but have a pH closer to the plasma pH.^(26,28)^ The infused liquid becomes distributed in approximately 30 minutes, and after this period, the increase in plasma volume is 50 to 75%.^(6)^

Colloids are fluids that contain macromolecules of sufficient weight to prevent their passage through a healthy endothelium. These fluids are classified as natural (albumin) or artificial (gelatins, dextrans, and hydroxyethyl starch [HES]).^(6,26)^

Colloids increase the plasma oncotic pressure, and due to the increased weight of the molecules, colloids remain within the vascular layer. While crystalloids equilibrate rapidly between compartments, in healthy endothelial barriers, colloids can remain in the intravascular space for up to 16 hours.^(6)^ The Saline *versus* Albumin Fluid Evaluation (SAFE) study involving more than 7,000 patients in Australia and New Zealand showed no difference in mortality at 28 days when comparing albumin to crystalloids.^(29)^

Gelatins, which are polypeptides derived from bovine collagen, have an albumin-like intravascular expansion capacity but are associated with increased renal damage.^(6)^ HES is a synthetic polymer derived from the replacement of amylopectin from sorghum, wax, or potatoes by a hydroxyethyl. Preparations with higher molecular weights are associated with higher rates of renal failure and coagulation disorders.^(6)^ The Crystalloid *versus* Hydroxyethyl Starch Trial (CHEST) study demonstrated a need for less fluid (30% less when compared to crystalloids), a faster elevation of central venous pressure (CVP), and a lower incidence of new shock but found a greater need for RRT in patients receiving HES.^(30)^ Studies comparing HES and crystalloids have also shown higher mortality in the group that received the synthetic polymer.^(6)^

## VOLUME RESUSCITATION

The resuscitation phase aims to restore the intravascular volume to promote the reversal of hypotension, increase in urinary output, normalization of pulses and peripheral perfusion, and improvement of the level of consciousness.^(31)^ Aggressive volume administration during fluid resuscitation may be associated with volume overload.^(5)^ The amount of fluid required to reverse shock at this stage is variable and unknown. Although rapid administration is associated with better outcomes, the response to this therapy should be evaluated.^(5,9,26)^ Volume management without adequate monitoring is a risk for volume overload.^(5)^ Management with vasopressor support should not be delayed, aiming at the restoration and maintenance of renal perfusion, optimizing diuresis, and avoiding fluid accumulation.^(1)^

Predicting the response to the infused volume reduces unnecessary fluid delivery. Monitoring of cardiac output and pulse pressure variation and evaluation of vena cava diameter and cardiac output by ultrasound are some tools used to verify the response of the patient to the administration of a volume bolus.^(32)^ These methods still have limitations due to the variations in the reference values in relation to the clinical stage of the patient. Some of these hemodynamic variables cannot be adequately measured in patients who are not ventilated or who are receiving small tidal volumes.^(26,33)^ However, the measurement of these variables, concomitant with passive lower limb elevation, may be useful in evaluating patients who are ventilating spontaneously.^(34)^ It is important to emphasize, however, that even if the patient responds to volume administration, it is not necessarily hypovolemic.^(35)^ In the case of hemodynamic instability, relative hypovolemia may occur due to vasoplegia induced by excessive sedatives or due to the infection process itself. In this situation, the more compliant venous layer favors blood stasis, culminating in an increase in hydrostatic pressure, further favoring the formation of edema and the leakage of fluid to the third space. Considering this presentation, the current recommendations for the treatment of septic shock propose that the use of vasoactive drugs in hypovolemic septic patients should not be delayed.^(1)^

Measures of central venous saturation and CVP were not shown to be sufficiently sensitive or specific to predict the response to fluid therapy.^(5,36)^ It is estimated that up to 50% of patients admitted to the ICU for sepsis do not respond adequately to these volume tests. In these cases, volume infusion only adds to the deleterious effects of volume overload.^(5,36)^ Markers of tissue hypoperfusion, such as lactate and central venous saturation, are generally used to assess the timing of discontinuation of resuscitation.^(37)^ A retrospective study of 405 septic patients who received treatment according to the target-guided therapy protocol based on central venous saturation, CVP, and mean arterial pressure (MAP) demonstrated a higher risk of FO and mortality.^(38)^ However, studies evaluating the use of continuous venous saturation as a marker of resuscitation response were more likely to find an association with volume overload.^(39)^

## MAINTENANCE VOLUME

In critically ill ICU patients, both hypervolemia and a cumulative fluid balance should be avoided. Thus, the treatment should be individualized for each patient and consider the clinical response during the resuscitation phase.^(9)^ As described, hypervolemia is associated with serious deleterious effects, with a higher risk of morbidity and mortality.^(36)^ The objectives of maintenance volume infusion are the preservation of intravascular volume and replacement of losses in progress, e.g., through drains, flow through intestinal fistulas, or probes.^(40)^ After reversion of hypotension, attention should be paid to adequate oxygen delivery (DO_2_) to the tissues, which is directly related to cardiac output, hemoglobin concentration, and arterial saturation.^(5,36)^

Conservative management of fluid administration in addition to initial resuscitation was associated with improved oxygenation rates, shorter MV time, and shorter hospital stay in patients with lung injury.^(12,21)^

Patients hospitalized in ICUs are constantly subjected to volume overload. In addition to the fluids received during the resuscitation phase, these patients receive a volume related to medications and nutrition, which easily promotes overload. Therefore, in this maintenance phase, it is important to minimize, or even avoid, the administration of non-essential fluids.^(9,40)^

Once FO is identified in patients with greater hemodynamic stability and reductions in vasopressors and MV parameters, the removal of excess volume should become a target, promoting a negative water balance.^(5,40)^

## IDENTIFY, PREVENT, AND TREAT HYPERVOLEMIA

Traditional indicators, such as MAP, heart rate, body weight, and peripheral edema, may not be reliable in critically ill patients. MAP and heart rate can be easily influenced by many factors, including the use of medications. Volume variables, such as end-diastolic volume and intrathoracic volume, may be useful but still require further study and clinical validation. Cardiac index and ejection fraction monitoring can be used to assess FO. In patients on MV, the absence of variations in pulse pressure may indicate FO. Chest radiography can also be a useful tool, e.g., through evaluation of Kerley B lines and engorgement of pulmonary vascularization.^(8)^

A study of 49 patients using the Doppler cross-sectional renal interlobar resistive index demonstrated a better ability to predict diuresis through the index than through the change in pulse pressure and the increase in MAP after volume administration, suggesting that renal hemodynamic improvement is essential for urinary output to occur.^(41)^

In septic patients with hypotension, the mechanism of renal autoregulation is impaired by the change in microcirculation, leading to organ failure.^(42,43)^ At this stage, vasopressor medications are frequently used in an attempt to maintain adequate renal perfusion pressure to preserve renal function and diuresis (Table 1). Studies in adults that analyzed the use of noradrenaline to maintain a MAP between 65 and 75mmHg demonstrated improvement in renal perfusion, with more favorable nephrological outcomes, better urine output, and less need for RRT. A randomized, double-blind clinical study comparing the use of low doses of norepinephrine with placebo in 40 children on MV using sedatives and analgesics showed an increase in blood pressure levels and a significant increase in diuresis in the group receiving norepinephrine.^(44)^ Therefore, to optimize renal perfusion pressure in patients in septic shock, noradrenaline has been an option.^(43,45)^ The target MAP during the treatment of septic shock is still not well established, and further studies are still needed. Evidence suggests that the target MAP must be individualized according to the patient history because very high blood pressure levels (e.g., 80 to 85mmHg) for previously healthy adults did not show benefits.^(42,43,45)^

**Table 1:** Main actions to prevent and treat fluid overload in critical patients

In the acute phase, carefully restore blood volume with isotonic fluids Avoid overestimating fluid maintenance or the use of hypotonic fluids In the presence of factors inducing vasoplegia (sedatives and opioids), aim to maintain the mean arterial pressure with vasoactive drugs (noradrenaline) while avoiding excess fluid infusions Mobilize limbs and change decubitus while avoiding gravitational fluid accumulation or lack of mobilization Aim for a more superficial sedation in patients on mechanical ventilation Neutral cumulative balance maintenance In patients with a positive cumulative balance, stimulate diuresis with low doses of intermittent diuretics (e.g., 0.2mg/kg furosemide) or continuous infusion In patients with deleterious effects (e.g., metabolic alkalosis) or patients who are refractory to furosemide, intravenous aminophylline may be combined with serum level control to increase diuresis Albumin administration would be indicated only in patients with a known cause of hypoalbuminemia In patients with impaired renal function and cumulative fluid balance, early renal replacement therapy should be planned

The therapeutic response to an infusion of loop diuretics, such as furosemide, depends on adequate renal perfusion, and there should be a minimal concentration of the medication at the site of action in the renal tubule. This concentration may be limited due to hypoalbuminemia or reduced renal perfusion. A dilution of sodium may also occur due to FO, even if the total body sodium is within the normal range or increased. In the case of low sodium concentrations in the distal portion of the loop of Henle, which is the site of action of loop diuretics, the therapeutic response will be lower than expected.^(46)^ For example, for a child receiving an intravenous maintenance volume of 70mL/kg/day, it would be necessary to achieve diuresis of at least 3mL/kg/hour to avoid a positive water balance. However, a critically ill patient receives much more than the ideal volume because in addition to the volumes from maintenance fluids and possibly nutritional compounds, the volumes from intermittent infusion of antibiotics and continuous vasoactive and sedoanalgesic medications are also added. Therefore, to successfully achieve the proposed water balance, it may be necessary at times to maintain a diuresis of approximately 5mL/kg/hr.

The use of loop diuretics, such as furosemide, has been shown to be effective in inducing diuresis in both children and adults. Low doses of diuretics (e.g., furosemide at 0.2mg/kg/dose) prevent episodes of acute hypovolemia. However, in patients with hemodynamic instability, continuous infusion of furosemide (0.1 to 0.3mg/kg/hour) can be used, which both ensures a continuous concentration of the medication at the site of action and avoids the compensatory mechanisms of sodium reabsorption between the doses of intermittent administrations. Blood volume oscillations, which have the possibility of hemodynamic worsening, are also avoided. With prolonged diuretic use, patients may develop resistance to the use of these medications.^(9)^ It is proposed to optimize the plasma concentration of the medication and to add other drugs to the therapy. In this situation, thiazide diuretics have also been shown to be effective in inducing diuresis. Blocking sodium reabsorption in other portions of the renal tubule avoids compensatory sodium reabsorption, increasing the efficacy of diuretics.^(46)^ Combinations with spironolactone and aminophylline have also been successful. A study that analyzed the effect of aminophylline on the induction of diuresis in 34 children up to 18 years of age found an average increase of 1.0mL/kg/hour (p = 0.0004) in the volume of diuresis after 24 hours.^(47)^ In patients with hypoalbuminemia consequent to the catabolism promoted by sepsis, the mobilization of fluid from the third space into the intravascular space may be impaired. Studies in adults have shown effective induction of diuresis, with improvement of oxygenation indexes and better control of water balance through combined infusion of albumin, followed by furosemide.^(9)^

The classic indications of RRT are FO, uremia, and electrolyte and metabolic disorders. Randomized clinical trials have suggested that early and continuous RRT in septic patients is associated with higher rates of renal recovery and lower mortality. Excessive delays at the onset of RRT are associated with unfavorable outcomes; however, the optimal time to initiate therapy is unclear.^(9,48)^

Water overload manifests clinically as edema, which also indicates excess fluid in the interstitium. The use of excessive sedation may lead to vasoplegia and hemodynamic instability, generating the need for more vasoactive medications and a higher risk of infusion of higher resuscitation volumes. Sedation and analgesia protocols, the use of pain and sedation scales, and the assistance of a trained multidisciplinary team have demonstrated the importance of adequate management of patients in terms of comfort, stress reduction, and the risk of withdrawal and delirium.^(49)^

Excess sedation also favors patient immobility, which is a currently known risk factor for critical patient neuromyopathy. Immobilization of critical patients is associated with microvascular dysfunction and fluid accumulation in the third space due to the increased hydrostatic pressure resulting from the decreased venous compliance, reduced pulmonary volume with increased risk of atelectasis, and increase in the pro-inflammatory products and byproducts of oxidative stress. Early mobilization studies have shown success in reducing delirium index, MV time, and length of stay in the ICU and hospital. This treatment has few adverse effects, with low rates of accidental extubation, accidents with falls, and episodes of transient desaturation.^(50)^

## CONCLUSION

Fluid overload, a potentially modifiable risk factor, is a frequent occurrence in pediatric intensive care unit. More severe clinical conditions require more aggressive resuscitations and thus a greater supply of fluid. The most recent evidence suggests deleterious effects of this overload. Monitoring strategies and volume offerings that consider the side effects of liberal use of volume in these patients may prevent potential complications, thus reducing the morbidity and mortality in the intensive care unit. Resuscitation should be individualized, and once hemodynamic stability has been achieved, volume overload should be promptly managed with diuretics or renal replacement therapy where indicated; with restriction of nonessential volumes, early mobilization, and adequate sedoanalgesia.
